# A multimodal approach for determining brain networks by jointly modeling functional and structural connectivity

**DOI:** 10.3389/fncom.2015.00022

**Published:** 2015-02-20

**Authors:** Wenqiong Xue, F. DuBois Bowman, Anthony V. Pileggi, Andrew R. Mayer

**Affiliations:** ^1^Boehringer Ingelheim Pharmaceuticals, Inc.Ridgefield, CT, USA; ^2^Department of Biostatistics, Mailman School of Public Health, Columbia UniversityNew York, NY, USA; ^3^Department of Biostatistics and Bioinformatics, Rollins School of Public Health, Emory UniversityAtlanta, GA, USA; ^4^The Mind Research Network/Lovelace Biomedical and Environmental Research InstituteAlbuquerque, NM, USA; ^5^Neurology Department, University of New Mexico School of MedicineAlbuquerque, NM, USA

**Keywords:** functional connectivity, structural connectivity, Bayesian analysis, small-world network, MCMC, fMRI, DTI, neuroimaging

## Abstract

Recent innovations in neuroimaging technology have provided opportunities for researchers to investigate connectivity in the human brain by examining the anatomical circuitry as well as functional relationships between brain regions. Existing statistical approaches for connectivity generally examine resting-state or task-related functional connectivity (FC) between brain regions or separately examine structural linkages. As a means to determine brain networks, we present a unified Bayesian framework for analyzing FC utilizing the knowledge of associated structural connections, which extends an approach by Patel et al. (2006a) that considers only functional data. We introduce an FC measure that rests upon assessments of functional coherence between regional brain activity identified from functional magnetic resonance imaging (fMRI) data. Our structural connectivity (SC) information is drawn from diffusion tensor imaging (DTI) data, which is used to quantify probabilities of SC between brain regions. We formulate a prior distribution for FC that depends upon the probability of SC between brain regions, with this dependence adhering to structural-functional links revealed by our fMRI and DTI data. We further characterize the functional hierarchy of functionally connected brain regions by defining an ascendancy measure that compares the marginal probabilities of elevated activity between regions. In addition, we describe topological properties of the network, which is composed of connected region pairs, by performing graph theoretic analyses. We demonstrate the use of our Bayesian model using fMRI and DTI data from a study of auditory processing. We further illustrate the advantages of our method by comparisons to methods that only incorporate functional information.

## 1. Introduction

There has been a recent emergence of complex functional brain network analyses. A typical neuroimaging network analysis involves defining brain regions (nodes) representing the whole brain, quantifying a measure of association between all pairs of brain regions to produce a connectivity matrix, thresholding these associations to obtain a more sparse connectivity matrix, and then calculating summary statistics that reflect properties of the network. Network summaries include metrics that reflect local or global communication ability (e.g., clustering coefficient, path length, and efficiency), centrality metrics (e.g., betweenness, closeness, and eigenvector centrality), and community structure (e.g., small-worldness).

A critical step in network analyses is that of quantifying associations, or functional connectivity (FC), between every pair of nodes because it fundamentally impacts the integrity, reliability, and interpretability of the resulting networks and their associated properties (Seghier and Friston, 2013; Ramsey et al., 2014). Friston et al. (1993) define FC as the “temporal correlations between spatially remote neurophysiological events.” This non-directional association may pertain to resting-state brain activity or to neural activity stemming from cognitive, emotional, visual, and behavioral tasks. In addition, estimation of directed connectivity is of great interest to describe graph theoretic properties in larger networks (Seghier and Friston, 2013). In contrast, structural connectivity (SC) refers to the structural white-matter fiber tracts (bundles of axons) linking different brain regions, which may be considered to identify structural brain networks. SC provides a mechanism for functional relationships in neural activity by enabling the transmission of electrical signals that pass along axons (Hendelman, 2000). Yet, FC and SC properties typically are evaluated separately.

The Pearson correlation coefficient and partial correlation are popular statistics used to describe nodal FC associations (Hampson et al., 2002; Marrelec et al., 2006). Some researchers use alternative measures of association, e.g., mutual information (Cover and Thomas, 1991), or consider transformations of the time series data, e.g., to examine associations in the frequency domain (Fiecas et al., 2010) or in the wavelet domain (Patel et al., 2006b). Patel et al. (2006a) develop a Bayesian model that assesses the FC based on the joint activation in pairs of brain regions. SC is based on diffusion tensor imaging (DTI), which is a magnetic resonance imaging (MRI) technique for characterizing the integrity of axonal fibers by measuring molecular diffusion (Kollias, 2009). Probabilistic diffusion tensor tractography (DTT) uses DTI data to empirically reconstruct fiber tracts by quantifying the likelihood of white-matter SC (Behrens et al., 2007).

A notable limitation of the aforementioned procedures for determining FC is that they do not consider any information about the associated SC, which may adversely impact the integrity of the networks used for complex whole-brain network analyses. Previous studies have suggested that structural fiber properties (degree of myelination and conduction times) contribute to FC amongst homolog brain regions (Stark et al., 2008; Zuo et al., 2010; Gee et al., 2011), but do not solely determine FC (Honey et al., 2009). There have been a few attempts to examine both FC and SC. These studies generally either examine the correspondence between SC and localized (voxel-level) analysis of functional MRI (fMRI) data or assess SC and FC sequentially and use SC to guide region selection for FC evaluation (or vice versa) (Rykhlevskaia et al., 2008). From such analyses, Morgan et al. (2009) suggest that FC is supported by SC along the language pathways. Also, Greicius et al. (2009) and van den Heuvel et al. (2009) indicate that resting-state FC reflects SC to a large degree. Bowman et al. (2012) present a framework that simultaneously considers fMRI and DTI data to determine FC, and they demonstrate that the supplemental SC information is particularly beneficial in the presence of fMRI noise. As the association between brain structure and function is revealed, an important next step is to develop unified, model-based statistical frameworks that incorporate both sources of information simultaneously. We feel that such methods will in turn improve the validity of complex network analyses.

We present a novel multimodal approach, using FC and supplementary SC information, to determine FC as the basis for defining and evaluating whole-brain networks. We extend the previously developed model by Patel et al. (2006a) that determines FC by examining the concurrence of elevated activity in pairs of brain locations. Our Bayesian model utilizes DTT information as a supplement to fMRI, and here we apply our model to evaluate FC globally between all pairs of defined regions of interest. We determine the hierarchy among functionally connected pairs of brain regions based on the associated probabilities of elevated activity for each node, giving rise to directed networks. We develop formal inference frameworks regarding task-related functional coherence for both our undirected and directed measures of association. Using the inference frameworks as a basis for thresholding, we build a functional network based on region pairs that are connected with high probability, and we explore the associated topological properties from the graphical network. We perform estimation using Markov Chain Monte Carlo (MCMC) techniques. We apply our method to an auditory spatial-cueing task data set and conduct simulation studies to evaluate the performance of our approach.

## 2. Data

### 2.1. Experimental data

Combined structural and functional data were collected from 32 right-handed adults (15 males, 17 females) on a 3 Tesla Siemens scanner. The functional data were collected during an auditory spatial-cueing task, which has been described in previous publications (Mayer et al., 2009a,b). The auditory stimuli consist of a series of two pure frequency tone pips per trial. The first auditory tone (2000 Hz) served as a spatial cue, and occurred with equal frequency in the left or the right ear. The second tone (1000 Hz) served as a target, and participants were instructed to press either their right index or middle finger for targets appearing in their left or right ear, respectively. Cues correctly predicted (i.e., valid trials) the location of the targets on 50% of the trials, with invalid trials (i.e., cue and target in opposite ears) occurring during the other 50% of trials. The stimulus onset asynchrony (SOA) between the cues and the targets was either 200 or 700 ms. Prior to the start of the experiment, participants were informed that the cues did not contain any useful information to predict the location of the target. Therefore, the cue validity ratio (50%) and task instructions were designed to evoke exogenous orienting conditions, with SOA designed to respectively measure facilitation (200 ms SOA) or inhibition of return (700 ms SOA). Participants were also required to maintain fixation on a centrally presented cross-hair to reduce the likelihood of eye movements. A total of 84 valid and 84 invalid trials were presented in pseudo-random order across three imaging runs. The main objectives considered here are to identify the functionally connected brain regions associated with the neural processing stemming from an auditory task (both valid and invalid trials), to determine underlying auditory networks, and to assess the topological properties of the networks, although a broader set of objectives has been previously considered (Mayer et al., 2009a,b).

### 2.2. Image acquisition and data preprocessing

High resolution 5-echo multi-echo MPRAGE T1 [TR (repetition time) = 2.53 s, 7° flip angle, number of excitations (NEX) = 1, slice thickness = 1 mm, FOV (field of view) = 256 mm, resolution = 256 × 256] and T2 [echo time = 77.0 ms, *TR* = 1.55 s, flip angle 152°, NEX = 1, slice thickness = 1.5 mm, FOV = 220 mm, matrix = 192 × 192, voxel size = 1.15 × 1.15 × 1.5 mm^3^] sequences were collected on a 3 Tesla Siemens Trio scanner. Echo-planar images (EPI) were collected using a single-shot, gradient-echo echoplanar pulse sequence [*TR* = 2000 ms; *TE* = 29 ms; flip angle = 75°; FOV = 240 mm; matrix size = 64 × 64]. A total of T^*^ = 483 fMRI scans were collected for each of the *N* = 32 subjects, 161 for each of three runs, after eliminating the first scan of each run to account for equilibrium effects. Two DTI scans with *b* = 800 s/mm^2^ and 30 diffusion gradients were acquired using a twice-refocused spin echo sequence to reduce the effects of eddy currents and artifacts associated with head movement and to allow increased time for diffusion sensitizing gradients. Additional DTI scanning parameters include the following: 72 interleaved slices, *TE* = 84 ms, *TR* = 9 s, flip angle = 90, slice thickness = 2.0 mm, FOV = 256 × 256 mm, matrix size = 128 × 128, voxel resolution = 2 × 2 × 2 mm^3^ (Ling et al., 2012).

We performed several standard preprocessing steps to the functional images using FMRIB (Functional Magnetic Resonance Imaging of the Brain) Software Library (FSL) (Smith et al., 2004). These steps included slice-timing correction, 3D motion correction and spatial normalization to Montreal Neurological Institute (MNI) space using a 12 degrees of freedom affine linear transformation with trilinear interpolation. Pre-whitening was conducted to remove the temporal correlations between scans from the same subject by iteratively estimating the autocorrelation matrix of the residuals to achieve independence through the whole time series (Woolrich et al., 2001).

### 2.3. Determining regional activity

We consider region-to-region connectivity in our network analysis. To define the brain regions, we begin with 90 regions from the automated anatomic labeling (AAL) system (Tzourio-Mazoyer et al., 2002) excluding the cerebellum. We further refine the regions by applying parcellation to obtain subregions within each AAL region as described in Appendix 1, which yields a total of 205 subregions. Each subregion that we consider contains more than 50 voxels. We identify the voxel within each subregion that is most involved with the auditory task, determined on the basis of a standardized statistic calculated by subtracting the mean and dividing by the standard deviation of each voxel time course. We summarize the neural activity for each defined subregion by selecting the roughly 150 closest voxels (in Euclidean distance) to the voxel most involved with the task, ensuring that these closest voxels all fall within the subregion. If there are fewer than 150 voxels in the subregion, we use the entire subregion. The area of the 150 voxels generally corresponds to a roughly spherical shape with a 6 mm radius, although we do not strictly require a spherical shape, e.g., to address boundary constraints. To obtain a single fMRI temporal profile representing each subregion for each subject, we perform a singular value decomposition (SVD) in the time domain to a T^*^ × 150 matrix. We extract the first right singular vector, yielding a single temporal profile reflecting the most dominant temporal trend in that subregion. Since singular vectors are unique up to multiplication by a unit phase factor, we compare the singular vector to the subregion mean signal to ensure that the selected signal represents the subregion correctly, and we apply a sign change to our extracted signal, if necessary. The resulting 205 temporal profiles reflect the neural activity representing each node in our network analysis.

### 2.4. Determining structural connectivity

We employ the widely used approach of Behrens et al. (2007), implemented in FSL, to perform probabilistic DTT. We define subregions within the AAL system for DTT, which are centered in white matter proximal to the fMRI-based subregions. Probabilistic tractography successively initiates streams, which are intended to follow or trace the paths of white matter tracts in the brain. A given number of streams (5000 in our analysis) are sent from the seed voxel, and each stream chooses a path based on the principle diffusion direction of the underlying white matter at each voxel and ceases according to a stopping rule. The probabilistic DTT for each pair of regions initially yields voxel-level counts (out of 5000 trials) indicating the likelihood of a fiber tract extending from the voxel in the seed region to (or through) the target region. We use the 90th percentile of the voxel-level counts connecting voxels in the seed region to voxels in the target region to reflect the strongest anatomical connectivity between pairs of regions. The voxel-level counts connecting two regions are usually asymmetric; yet for our purpose, we regard FC as a symmetric measure. Therefore, we impose symmetry of SC between two regions by calculating the maximum of the two directional counts for each region pair. To reduce the noise, we analyze both DTI scans and average the resulting SC counts. We adjust the SC counts by the corresponding geometric distances between regions by fitting a zero-inflated Poisson regression model on voxel-level SC counts adjusted for the minimum geometric distance between regions.

## 3. Methods

**Figure 1:**
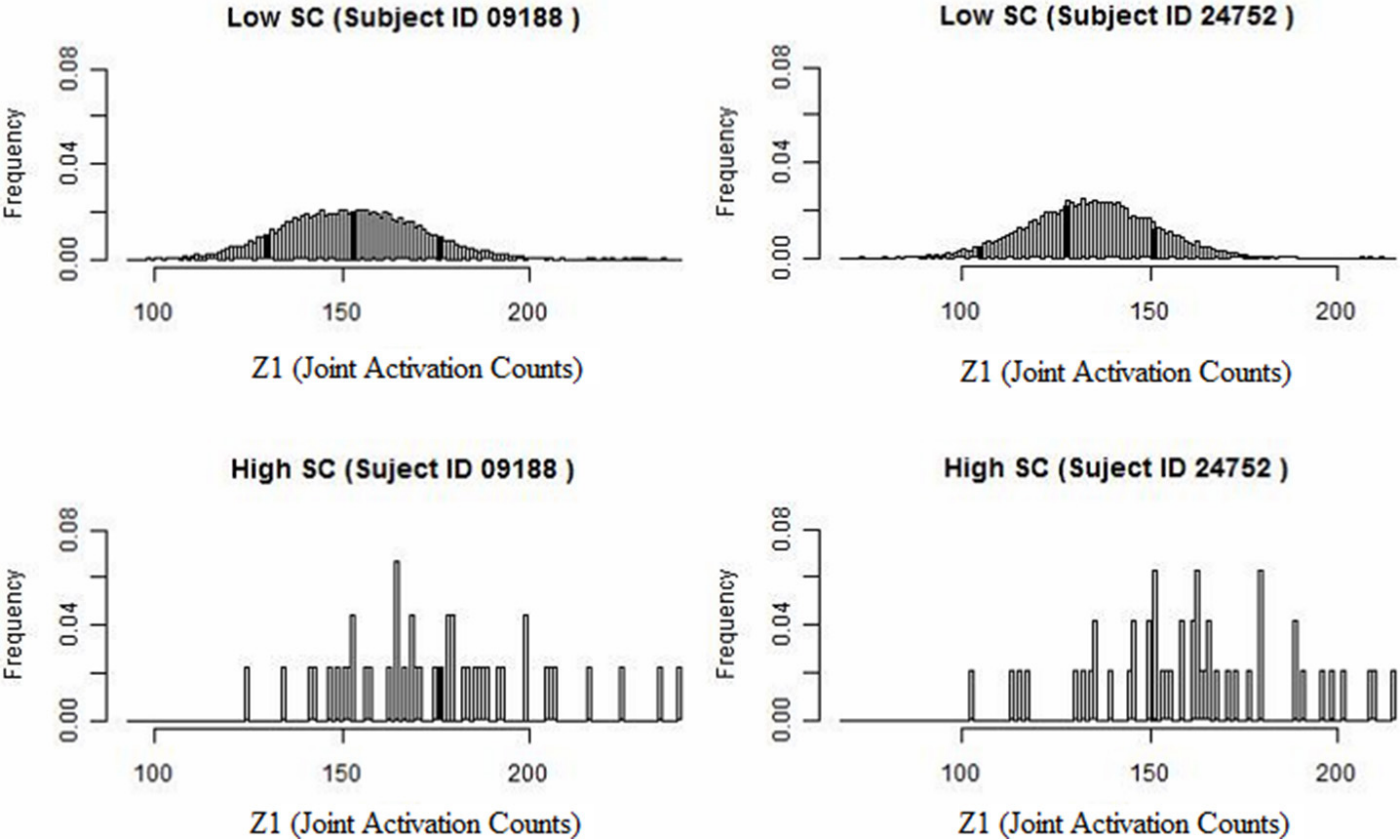
**Histogram of joint activation counts (*Z*_1_) at a lower and higher level of structural voxel-level counts for two subjects.** Note that the joint activation values tend to be larger between region pairs exhibiting high SC relative to low SC.

We develop a framework for network analysis, which jointly considers FC and SC. We introduce a statistic κ to capture the functional coherence between region pairs and an associated ascendancy measure τ to quantify the hierarchy of identified coherent regions. The way that we describe functionally coherent brain regions is not a typical definition of FC. The joint activation framework is defined conceptually by Patel et al. (2006a). This method performs well-relative to other methods for FC and comparisons conducted by Smith et al. (2010) and by Ramsey et al. (2014), which considers performance after removing and replacing the high-pass filter by a less stringent filter. A preliminary look at our data reveals that higher levels of SC counts tend to have associated larger values of functional coherence (see Figure 1). Specifically, we examine the distribution of functional coherence at lower and higher levels of voxel-level SC for each subject across 20910 region pairs based on 205 brain regions. Figure 1 illustrates the results for selected subjects, but the results across all the subjects reveal similar patterns. Therefore, we build our Bayesian model based on the observation that increased SC is generally associated with higher functional coherence. SC is a static property, whereas FC is a transient characteristic that may vary with the performance of different tasks. Therefore, we do not make the link too strong in our model since high SC may exist without corresponding elevated FC during the auditory task.

### 3.1. Joint activation and structural connectivity

Define *A*_*gnt*_ = *I*(*R*_*gnt*_ > *c* × σ_*gn*_), where *R*_*gnt*_ = *Y*_*gnt*_ − $\hat{\mu }$_*gn*_ is the mean-adjusted level of neural activity for region *g*, subject *n*, and scan *t*; *c* is a constant; $\hat{\mu }$_*gn*_ is the mean of the subregion level fMRI profile *Y*_*gnt*_ across time, and σ^2^_*gn*_ is the variance of *Y*_*gnt*_, with *n* = 1, …, *N* and *t* = 1, …, T^*^. Thus, *A*_*gnt*_ serves as an indicator of elevated regional brain activity at time *t*. We choose *c* = 0.01 when analyzing the auditory spatial-cueing task.

The joint activation between two regions *a* and *b* for subject *n* can be expressed as:

(1) $$\begin{array}{l} Z_{1}^{*}=\text{​}\sum_{t\;=\;1}^{T^{*}}I(A_{ant}=1,A_{bnt}=1),\;Z_{2}^{*}=\text{​}\sum_{t=1}^{T^{*}}I(A_{ant}=1,A_{bnt}=0)\\ Z_{3}^{*}=\text{​}\sum_{t=1}^{T^{*}}I(A_{ant}=0,A_{bnt}=1),\;Z_{4}^{*}=\text{​}\sum_{t=1}^{T^{*}}I(A_{ant}=0,A_{bnt}=0). \end{array}$$

Z^*^_1_ is interpreted as the number of times that both regions *a* and *b* experience an elevated fMRI signal (for subject *n*). Note that we omit subscript indexing subjects to simply our notation. We assume Z^*^ = (Z^*^_1_, …, Z^*^_4_)′ follows a multinomial distribution with parameters T^*^ and θ = (θ_1_, …, θ_4_)′, where

(2) $$\begin{array}{l} \theta_{1}=P\left(A_{ant}=1,A_{bnt}=1\right),\;\theta_{2}=P\left(A_{ant}=1,A_{bnt}=0\right)\\ \theta_{3}=P\left(A_{ant}=0,A_{bnt}=1\right),\;\theta_{4}=P\left(A_{ant}=0,A_{bnt}=0\right). \end{array}$$

To facilitate interpretations across different analyses, we standardize Z^*^_i_ (*i* = 1, …, 4) by scaling it with a specified number of scans. We set the scaling unit to *T* = 100 for our data, so our standardized measure, *Z*_*i*_, is the average number of times that *a* and *b* are coherent per one hundred scans. **Z** also follows a multinomial distribution with parameters *T* and θ.

For the anatomical data, we denote the DTT counts between regions *a* and *b* for subject *n* by S^*^ (omitting the subject and region subscripts for simplicity). Let M^*^ be the number of trials for probabilistic DTT fiber tracking from the voxels in the seed region. We assume that S^*^ follows a binomial distribution with parameters M^*^ and π, where π is the probability of SC between regions *a* and *b* for any subject. Using similar scaling applied to Z^*^_i_, we generate scaled counts *S*, which follow a binomial distribution with parameters *M* and π, where here we choose *M* = 1000.

To estimate the model parameters, we perform 10,000 iterations proceeded by 2000 burn-in iterations. The programming is implemented in Matlab, and the computation is performed on a Linux cluster with 16 GB of RAM. Execution time is approximately 3–4 h. Additional gains in computation time may be achievable by parallel programming. In simulation studies, 7000 iterations proceeded by 2000 burn-in iterations are conducted for each simulated dataset. For both our data analysis and simulations, we implement a thinning procedure by retaining every tenth iteration from the MCMC chain for easy storage and for promoting independence between the samples. A total of 10,000 datasets are generated for each combination of hyperparameters and each job is completed within 1 day.

### 3.2. Functional coherence and ascendancy

We extend the agreement measure of Patel et al. (2006a), which evaluates joint activation only (see Appendix 2) based on Cohen's Kappa (Cohen, 1960), to describe functional coherence between pairs of brain regions. Considering Table 1, our functional coherence measure κ is defined as:

(3) $$\kappa =\{\begin{array}{ll} \frac{\theta_{1}+\theta_{4}-E}{1-E} & \text{if }\theta_{1}\theta_{4}>\theta_{2}\theta_{3}\\ 0 & \text{otherwise}, \end{array}$$

where *E* = (θ_1_ + θ_2_)(θ_1_ + θ_3_) + (θ_3_ + θ_4_)(θ_2_ + θ_4_). The numerator of κ measures the difference between the probability of coherence and the expected probability of coherence under independence. We restrict our attention to non-negative values of κ, so our measure of agreement ranges from 0 to 1. κ equals 1 when the probability of joint activation and deactivation θ_1_ and θ_4_ sums to 1, and hence θ_2_ and θ_3_ are 0, which indicates complete coherence. If there is no agreement between regions *a* and *b*, κ = 0.

**Table 1:** **Joint activation probabilities for regions *a* and *b***.

		**Region a**		
		**Active**	**Inactive**	
**Region b**	**Active**	θ_1_	θ_3_	θ_1_ + θ_3_
	**Inactive**	θ_2_	θ_4_	θ_2_ + θ_4_
		θ_1_ + θ_2_	θ_3_ + θ_4_	1

Given that *a* and *b* are functionally connected, i.e., κ exceeds a specified threshold (say *e*_κ_) with high probability, we define a measure of ascendancy to determine the hierarchical relationship between the regions. Unlike the definition in Patel et al. (2006a), in which the definition of ascendancy is based on the ratio of *P*(*A*_*a*_ = 1) and *P*(*A*_*b*_ = 1) (see Appendix 2), our ascendancy measure, τ_*ab*_, is based on the ratio of *P*(*A*_*a*_ = 1)/(1 − *P*(*A*_*a*_ = 1)) and *P*(*A*_*b*_ = 1)/(1 − *P*(*A*_*b*_ = 1)) and takes the following form:

(4) $$\tau_{ab}=\left(\frac{\theta_{1}+\theta_{2}}{\theta_{3}+\theta_{4}}\right)/\left(\frac{\theta_{1}+\theta_{3}}{\theta_{2}+\theta_{4}}\right).$$

**Figure 2:**
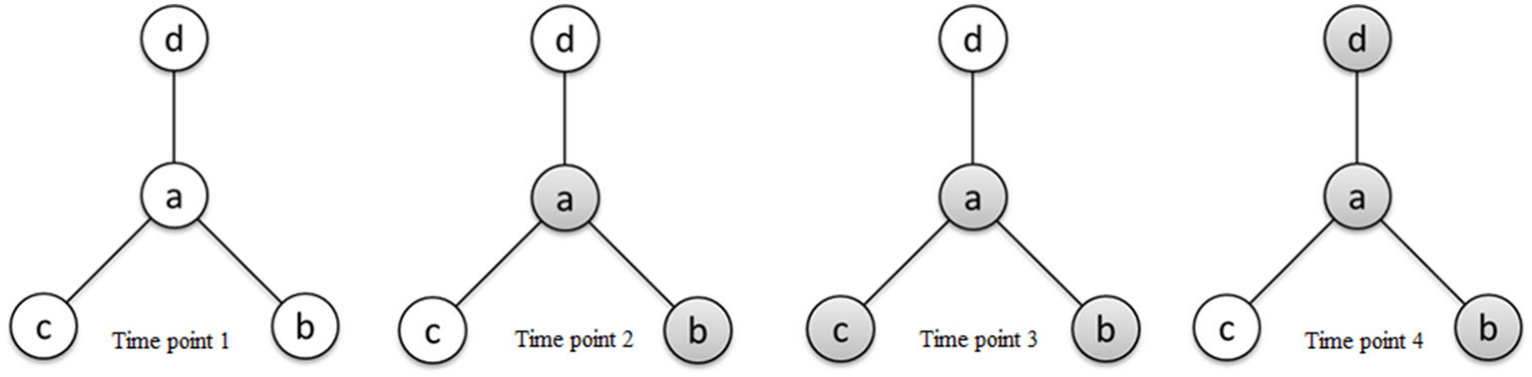
**Schematic diagram of activity states of four regions *a*, *b*, *c*, and *d* at different time points and connectivity relationships between the regions reflecting functional connectivity and ascendancy.** Shading for a given region indicates elevated activity at the corresponding time point. The line segments connecting regions define functionally connected region pairs, illustrating that region *a* is functionally connected to regions *b*, *c*, and *d*. Region *a* is ascendant to regions *c* and *d* based on the relative levels of elevated activity between functionally connected region pairs.

τ_*ab*_ ranges from 0 to ∞ and is interpreted as the odds of region *a* being active relative to the odds of region *b* being active. If two regions *a* and *b* become active together and inactive together, we consider them as functionally connected; however, an ascendancy relationship does not necessarily exist between them. Given that region *a* and region *b* are functionally connected, we say that *a* is ascendant to *b* whenever the marginal odds of activation of *a* are larger than that of *b*. Given κ > *e*_κ_, τ_*ab*_ > 1 indicates that *a* is ascendant to *b*, while τ_*ab*_ < 1 indicates that *b* is ascendant to *a*. Figure 2 presents a hierarchical relationship among four regions. We use shading to denote a region exhibiting elevated activity. As shown in Figure 2, while region *a* is functionally connected to regions *b*, *c*, and *d* (represented by solid lines in the graph), regions *c* and *d* exhibit elevated activity for a strict subset of the stimuli which *a* exhibits elevated activity, suggesting that *a* is ascendant to *c* and *d*.

### 3.3. Bayesian statistical model

For any pair of regions *a* and *b*, the likelihood function takes the form:

(5) $$p(Z,S|\theta ,\pi )\propto \prod_{i=1}^{4}\theta_{i}^{\sum_{n=1}^{N}Z_{in}}\pi^{\sum_{n=1}^{N}S_{n}}{\left(1-\pi \right)}^{N\times M-\sum_{n=1}^{N}S_{n}},$$

where the proportional sign (∝) defines an equivalent relation up to a multiplicative constant. Following the approach by Patel et al. (2006a), we assume that each repeated measure on the same region pair is independent across subjects. We also assume that each repeated measure on the same region pair is independent over time since we have performed pre-whitening in our pre-processing to remove the temporal correlations between scans from the same subject. In addition, given both probability measurements **θ** and π, *S* is independent of **Z** because we build structure-function dependence in the distribution of [**θ**|π]. This is a conditional independence assumption between *S* and **Z**, but marginally our model still captures the dependence between *S* and **Z** through the corresponding parameters **θ** and π.

Using a Bayesian formulation, we express our prior belief about structural connection probabilities π by defining a beta prior which takes the form:

(6) $$p(\pi )\propto \pi^{\alpha_{0}-1}{\left(1-\pi \right)}^{\beta_{0}-1}.$$

We choose the beta distribution as the prior both for mathematical convenience, as it is the conjugate prior for the binomial distribution, and for its flexibility to implement priors with different shapes of the density using various hyper prior parameter specifications.

We specify a flat prior for our DTT data by setting α_0_ = β_0_ = 1 for each region pair, suggesting no available prior information regarding the SC of any region pair. In our simulation studies, we evaluate the performance of our method under different combinations of the hyperparameters α_0_ and β_0_.

The prior for **θ** is taken to follow a Dirichlet distribution with parameters (α(π) + α_1_, α_2_, α_3_, α_4_)′, where α(π) is a function of π and reflects the assumed relationship between FC and SC. Specifically, we assume that

(7) $$\begin{array}{l} p(\theta |\pi )\propto \frac{\Gamma (\alpha (\pi )+\alpha_{1}+\alpha_{2}+\alpha_{3}+\alpha_{4})}{\Gamma (\alpha (\pi )+\alpha_{1})}\\ \;\times \theta_{1}^{\alpha (\pi )+\alpha_{1}-1}\theta_{2}^{\alpha_{2}-1}\theta_{3}^{\alpha_{3}-1}\theta_{4}^{\alpha_{4}-1}. \end{array}$$

We set α_1_ = 5, α_2_ = α_3_ = α_4_ = 10, and $\alpha (\pi )=10/\left(\frac{9}{\text{l}n(\text{10})}-1\right)\times 10^{\pi }-10/\left(\frac{9}{\text{l}n(\text{10})}-1\right)$, so the average value of α(π) on π ∈ [0, 1] is 10. In this case, θ_2_, …, θ_4_ have the same expected values and moderate variances. Our prior is based on the observation that weak SC corresponds to relatively few joint functional activations, and extremely strong SC is assumed to yield an expected value of θ_1_ to be around 0.5, which is approximately the highest maximum likelihood estimate of θ_1_. When α(π) is an increasing function, the expected value of θ_1_, which takes the form of (α(π) + α_1_)/(α(π) + α_1_ + α_2_ + α_3_ + α_4_) is also an increasing function with respect to π; thus, matching our observation from the data. Later, we present results from a sensitivity analysis of our choice of α(π), which shows that our results do not change much with respect to different functions of α(π).

### 3.4. Sampling the joint posterior probability distribution

We express the posterior probability distribution from our Bayesian model as:

(8) $$\begin{array}{l} p(\theta ,\pi |Z,S)\propto \frac{\Gamma (\alpha (\pi )+\alpha_{1}+\alpha_{2}+\alpha_{3}+\alpha_{4})}{\Gamma (\alpha (\pi )+\alpha_{1})}\\ \;\times \pi^{\sum_{n\;=\;1}^{N}S_{n}+\alpha_{0}-1}{\left(1-\pi \right)}^{N\times M-\sum_{n\;=\;1}^{N}S_{n}+\beta_{0}-1}\\ \;\times \theta_{1}^{\alpha (\pi )}\prod_{i\;=\;1}^{4}\theta_{i}^{\sum_{n\;=\;1}^{N}Z_{in}+\alpha_{i}-1}. \end{array}$$

The full conditional posterior distributions take the form:

(9) $$\begin{array}{l} p(\pi |\theta ,Z,S)\propto \frac{\Gamma (\alpha (\pi )+\alpha_{1}+\alpha_{2}+\alpha_{3}+\alpha_{4})}{\Gamma (\alpha (\pi )+\alpha_{1})}\\ \;\times \pi^{\sum_{n=1}^{N}S_{n}+\alpha_{0}-1}{\left(1-\pi \right)}^{N\times M-\sum_{n=1}^{N}S_{n}+\beta_{0}-1}\theta_{1}^{\alpha (\pi )} \end{array}$$

and

(10) $$\begin{array}{l} p(\theta |\pi ,Z,S)\sim \text{Dirichlet}(\text{​​}\sum_{n=1}^{N}Z_{1n}+\alpha (\pi )+\alpha_{1},\sum_{n=1}^{N}Z_{2n}+\alpha_{2},\\ \;\sum_{n=1}^{N}Z_{3n}+\alpha_{3},\sum_{n=1}^{N}Z_{4n}+\alpha_{4}). \end{array}$$

Estimation of κ and τ are based on the posterior distribution, *p*(**θ**|**Z**, *S*), as we are able to estimate *p*(κ|**Z**, *S*) and *p*(τ|**Z**, *S*) by sampling *p*(**θ**, π|**Z**, *S*). We set effect sizes for κ, denoted *e*_κ_, and τ, denoted *e*_τ_, in our analysis to compute exceedance probabilities *P*(κ > *e*_κ_ | **Z**, *S*) and *P*(τ > *e*_τ_ | **Z**, *S*) from our modeling framework. For κ, we choose *e*_κ_ = 0.4, which reflects moderate agreement or above (Landis and Koch, 1977). As no standard has been established for hierarchy, we explore the ascendancy relationship when τ is above 50th percentile across all region pairs. We conduct inference on κ and τ by estimating *P*(κ > *e*_κ_ | **Z**, *S*) > *p*_κ_ and *P*(τ > *e*_τ_ | **Z**, *S*) > *p*_τ_, respectively. We set both *p*_κ_ and *p*_τ_ to 0.5 to capture the most information. The choices of effect sizes and probability thresholds are made to reflect characteristics of functional coherence and ascendancy, rather than on the basis of statistical properties. Ideally, these thresholds should be determined before performing the data analysis. However, due to the complexity and variability of the neuroimaging data, the user has the flexibility to investigate connections between regions at different levels of these thresholds.

As revealed by the conditional posterior distribution of θ, our prior belief has an impact on the posterior through α(π), but does not drive the direction of results. As the SC π becomes stronger, the expected value of θ_1_ increases, and the expected values of θ_2_, …, θ_4_ decrease accordingly, but at a slower rate.

We estimate our Bayesian hierarchical model using MCMC methods, implemented via the Gibbs sampler with an embedded Metropolis step. The parameter **θ** is updated from a Dirichlet distribution with π specified from the previous step. The parameter π is updated by π^*^, which is sampled from a Normal jumping distribution *J*_*t*_(π^*^|π^*t*−1^) = *N*(π^*^|π^*t* − 1^, τ^2^) at time *t*, with probability min (*r*, 1), where *r* is the ratio of the conditional densities from time *t* − 1 to the conditional densities of the proposed value with respect to **θ**,

(11) $$r=\frac{p(\pi^{*}|\theta ,Z,S)}{p(\pi^{t-1}|\theta ,Z,S)}.$$

The variance τ^2^ in the Normal jumping distribution is adjusted based on the data, which ensures the acceptance ratio close to 25% to achieve the optimal efficiency of Metropolis algorithm Gelman et al. (1995).

### 3.5. Assessing brain networks

We build both a undirected and a directed brain network based on the coherence and ascendancy relationships between regions, respectively, and perform graph theoretic analyses to demonstrate topological properties of the brain networks. Suppose there are *s* brain regions and *t* functionally connected region pairs in the network, which are represented by *s* nodes and *t* edges, respectively. In a directed network, if region *a* is ascendant to region *b*, the edge between them is directed from region *a* to region *b*.

Network topology is described as a small-world network if compared to a similar random network, the small-world index σ = (*C*/*C*_random_)/(*L*/*L*_random_) > 1 (Watts and Strogatz, 1998; Humphries et al., 2006). Here, a similar random network is defined as a network with the same number of nodes, the same number of edges, and the same degree distribution (Simpson et al., 2013). The clustering coefficient *C* measures the average likelihood of connecting neighbors. The clustering coefficient is defined as *C*_*i*_ = 2*E*_*i*_/*k*_*i*_(*k*_*i*_ − 1) for undirected network, and *C*_*i*_ = *E*_*i*_/*k*_*i*_(*k*_*i*_ − 1) for directed network for each node *i*, where *k*_*i*_ is the number of neighbors of node *i* and *E*_*i*_ is the number of direct links connecting neighbors of node *i*. And the path length *L* is the average minimum number of connections to link two nodes.

We also examine the hubs in the network as they play a central role in a network since they serve as the common connections to other nodes. We define hubs as the nodes with degree (*D*), out-degree (*D*_*out*_) or in-degree (*D*_*in*_) at least one standard deviation above the mean degree of all the nodes in the network (Sporns et al., 2007). For directed networks, driving hubs satisfy *D*_*out*_ > mean + SD and driven hubs satisfy *D*_*in*_ > mean + SD. Roughly speaking, hubs are regions that exhibit numerous connections to other regions, with driving hubs, being relatively more active than the regions to which they are connected; and driven hubs, being relatively less active than the regions to which they are connected.

## 4. Results

### 4.1. Auditory data results

We apply our Bayesian model to the auditory processing fMRI data to determine functionally connected regions and also to examine the corresponding coherence and ascendancy relationship to illustrate the neural integration underlying auditory and related processing. We find strongly connected regions within the auditory cortex with associated hierarchical relationships between these functionally connected pairs of regions.

We identify an undirected network based on the region pairs with *P*(κ > *e*_κ_ | **Z**, *S*) > *p*_κ_. We determine the hubs in the network using the criteria described in Section 3.5. A total of 27 hubs are found, including brain regions within the auditory cortex, visual cortex, motor cortex, and sensory regions. For example, as an important region in the auditory cortex, the left superior temporal gyrus plays a central role in the undirected network. Also, the bilateral Rolandic operculum, which is one of the auditory-speech encoding regions, shares many connections with other regions, which may reflect the use by many people of language encoding areas during auditory processing involving perception of the spatial location of a tone (Klatzky et al., 2002). The design of the spatial-cueing auditory experiment calls for subjects to momentarily remember the location of the target tone before they press the button. Many people make use of visualization to aid the memory process, especially spatial memory, which may in turn activate the visual cortex (Ungerleider, 1995). Our findings support this relationship by revealing the prominent roles of superior parietal gyrus, the left precuneus, and other regions within the visual cortex in the network. In addition, our results reflect the neural processing related to button presses in response to the target cue by identifying the bilateral supplementary motor area, the right precentral gyrus, and the left postcentral gyrus as hubs, all of which are included in motor and sensory systems. We further examine other graph theoretic properties of the brain network associated with the auditory task. The clustering coefficient *C* of this undirected network is 0.02, which reflects a low likelihood of connected neighbors in the network. However, the path length *L* is 3.24, which indicates that it takes an average of only three intermediate connections to link any pair of regions in the network. The identified undirected network has the small-world property with the small-world index $\hat{\sigma }$ = 35.16 compared to the average of 1000 similar random networks (*C*_*random*_ = 8.04 × 10^−4^, *L*_*random*_ = 4.58).

**Figure 3:**
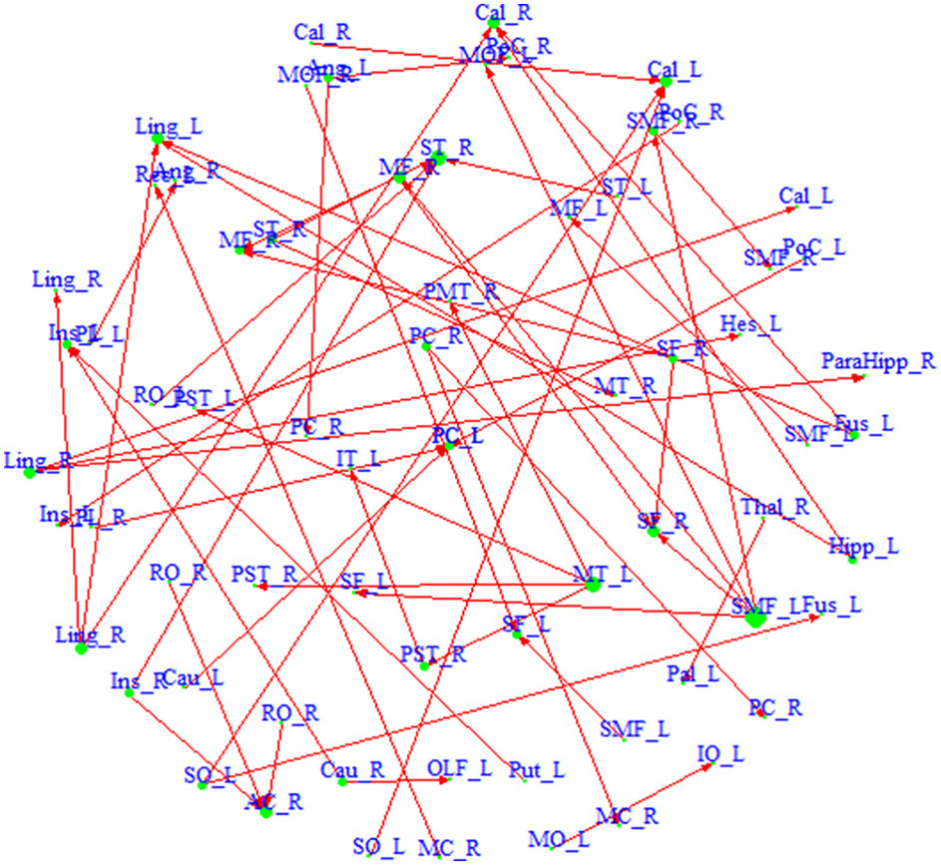
**A directed network based on region pairs with *P*(κ > 0.4 | **Z**, *S*) > 0.5 and *P*(τ > *e*_τ_ | **Z**, *S*) > 0.5.** An arrow from region *a* to region *b* means that region *a* is ascendant to *b*. Size of each region represents its degree. Driving hubs have more connections directed to other regions, e.g., SMF_L; and driven hubs have more connections directed to itself, e.g., ST_R. A list of the regions included in the network is available in Appendix 3.

We also build a directed network based on the region pairs with *P*(κ > *e*_κ_ | **Z**, *S*) > *p*_κ_ and *P*(τ > *e*_τ_ | **Z**, *S*) > *p*_τ_ (Figure 3). Similarly, both driving hubs and driven hubs are identified. Here, we focus on the driving hubs, since they are the regions that play more central roles when connecting to others. With the constraint of ascendancy, substantially fewer hubs are found compared to the undirected network. We identify a total of seven driving hubs, including the right Rolandic Operculum, the left olfactory gyrus, the left supplementary motor area, the right middle cingulate gyrus, the left superior occipital gyrus, the right middle temporal gyrus and the left superior medial frontal gyrus (component figures for each driven hub are shown in Appendix 4). These driving hubs are more active than the other regions to which they are functionally connected. The clustering coefficient *C* of the directed network is 0.26, and the path length *L* is 4.17. The directed network has higher probability of connecting neighbors comparing to undirected network, and it takes about four steps to connect any pair of regions within the network. Compared to the average of 1000 similar random networks (*C*_*random*_ = 0.02, *L*_*random*_ = 4.71), the identified directed network has the small-world index $\hat{\sigma }$ = 12.93, which indicates that it demonstrates small-worldness.

For comparison, we apply the approach of Patel et al. (2006a) to fMRI data from the auditory spatial cueing task. Their method only utilizes functional imaging data and is limited to using joint activation to define connectivity and ascendancy. The Patel method fails to identify the left superior temporal gyrus as a hub in the undirected network, which is an important region within the auditory cortex. Another major difference is that more region pairs within the visual cortex are detected by the original approach. Although visualization is an essential component of the experimental design, we do not expect to see the high degree of connectivity within the visual cortex because the task only involves fixation on a cross hair, with more engaging auditory processing. This leads us to conjecture that—without structural information, more false positives are generated while the major findings may go undetected.

### 4.2. Simulation results

We conduct two simulation studies to compare our approach to alternative methods that solely consider FC, without regard for underlying SC. Specifically, we compare our method to the approach of Patel et al. (2006a) and to a traditional correlation-based analysis. In addition, we conduct a analysis to evaluate the sensitivity of our approach to the impact of various choices of the function α(π), which links FC and SC.

We first discuss simulation results from the comparison our combined fMRI and DTI approach (labeled as the *FC with SC* method) with the approach by Patel et al. (2006a) utilizing fMRI data only (labeled as the *FC only* method). We generate data *Z* and *S* from our model with different settings of hyperparameters α_0_ and β_0_ in the prior distribution of π. These prior parameters control the shape of the density function and provide a flexible range of possible prior distributions. We evaluate the methods by comparing the bias of the corresponding posterior means of **θ**, κ and τ. For each simulation setting, 10 π's are generated, and 10 θ's are simulated for each π, 100 data sets are generated from each set of θ. Therefore, a total of 10,000 data sets are simulated to compute the mean biases. The results indicate that our method performs better in all settings with smaller bias. Table 2 shows that the mean bias of θ_*i*_'s from our method is smaller than that from *the FC only* method in every case, which indicates that incorporating structural information improves the estimation of FC. Since our definitions of κ and τ are both functions of θ_*i*_'s, it follows that our model also outperforms *the FC only* method for estimating these measures as defined in (3) and (4). Alternatively, we compare estimation performance of κ and τ from *the FC only* method based on the original definitions from Patel et al. (2006a) with estimation of our extended definitions of κ and τ under our *FC with SC* approach. We contrast how these two methods address functional coherence/association and ascendancy. The standard deviation of the bias also yields similar conclusions favoring our combined *FC with SC* approach over *the FC only* method (see Table 4 in Appendix 5). We examine the performance of our method using samples sizes of 15, 30, and 100 subjects. Although the difference in bias between the two approaches is relatively small, our FC with SC approach outperforms the FC only approach in every case that we consider.

**Table 2:** **Comparison of mean of bias between two Bayesian methods**.

**α_0_**	**β_0_**	**E(π)**	**FC with SC(× 10^−3^)**						**FC only(× 10^−3^)**					
			**θ_1_**	**θ_2_**	**θ_3_**	**θ_4_**	**κ**	**τ**	**θ_1_**	**θ_2_**	**θ_3_**	**θ_4_**	**κ**	**τ**
***N*** = 15														
1	100	0.01	6.882	8.906	9.006	9.078	2.874	44.578	6.951	9.011	9.154	9.183	221.313	106.883
2	18	0.1	7.39	9.073	8.851	8.822	4.442	42.553	7.461	9.162	8.993	8.914	204.43	122.885
2	5	0.3	8.565	8.861	8.544	8.58	8.545	41.804	8.667	9.038	8.636	8.689	120.509	126.061
2	2	0.5	9.239	8.333	8.484	8.499	13.308	41.421	9.297	8.44	8.63	8.644	71.363	128.054
5	2	0.7	9.798	8.146	8.086	8.196	16.415	40.797	9.862	8.301	8.174	8.316	59.667	136.606
18	2	0.9	10.056	7.379	7.52	7.313	19.624	41.791	10.233	7.503	7.68	7.457	88.121	136.543
***N* = 30**														
1	100	0.01	4.925	6.550	6.394	6.391	2.185	30.759	4.963	6.587	6.432	6.416	233.875	116.591
2	18	0.1	5.127	6.361	6.282	6.469	2.791	29.577	5.173	6.427	6.307	6.508	188.989	117.824
2	5	0.3	5.979	6.292	6.206	6.211	5.702	29.458	6.017	6.330	6.247	6.247	116.983	127.239
2	2	0.5	6.271	6.044	6.045	5.952	7.556	30.224	6.321	6.106	6.077	5.998	112.727	119.231
5	2	0.7	7.007	5.777	5.653	5.692	12.085	29.761	7.078	5.829	5.711	5.756	57.719	123.946
18	2	0.9	7.150	5.387	5.330	5.390	13.531	28.887	7.242	5.434	5.376	5.442	80.766	139.150
***N* = 100**														
1	100	0.01	2.704	3.536	3.597	3.534	1.120	17.098	2.710	3.541	3.597	3.545	232.029	115.003
2	18	0.1	2.996	3.483	3.481	3.532	2.103	16.705	3.006	3.492	3.485	3.538	181.038	114.898
2	5	0.3	3.139	3.435	3.377	3.380	2.717	16.048	3.150	3.446	3.389	3.385	153.390	126.220
2	2	0.5	3.540	3.362	3.266	3.315	4.950	16.250	3.541	3.362	3.273	3.327	93.892	127.077
5	2	0.7	3.748	3.172	3.122	3.066	6.080	15.228	3.761	3.177	3.131	3.077	78.071	161.000
18	2	0.9	3.939	2.938	3.024	2.920	7.495	16.320	3.959	2.949	3.037	2.931	84.696	144.013

*The table reveals the improvements of FC with SC from FC only in terms of the mean of bias*.

Our second simulation study compares our method to a traditional correlation analysis. We use the same simulated **θ** from the previous simulation study to generate the neural activity profiles *Y*_*ant*_ and *Y*_*bnt*_, for regions *a* and *b*, respectively, from a bivariate normal distribution with variances σ^2^_*a*_ and σ^2^_*b*_ and correlation ρ. Thus, the mean adjusted level of neural activity profiles *R*_*ant*_ and *R*_*bnt*_ also follow a bivariate normal distribution. We derive the expectation of *Z*_*i*_'s as follows:

(12) $$\begin{array}{l} E(Z_{1})=P(Z_{1}=1)=P(R_{ant}>c_{a},R_{bnt}>c_{b})=\theta_{1}\\ E(Z_{2})=P(Z_{2}=1)=P(R_{ant}>c_{a},R_{bnt}<c_{b})=\theta_{2}\\ E(Z_{3})=P(Z_{3}=1)=P(R_{ant}<c_{a},R_{bnt}>c_{b})=\theta_{3}\\ E(Z_{4})=P(Z_{4}=1)=P(R_{ant}<c_{a},R_{bnt}<c_{b})=\theta_{4}. \end{array}$$

We solve for *c*_*a*_ and *c*_*b*_ using the marginal probabilities of *R*_*ant*_ and *R*_*bnt*_, which are functions of **θ**, and we subsequently solve for ρ using any of the above equations. We estimate the Pearson correlation coefficient ρ from our simulated data and compare it to the functional coherence κ. We expect to see substantial correspondence between these two measures since they both capture the functional associations between two regions.

**Figure 4:**
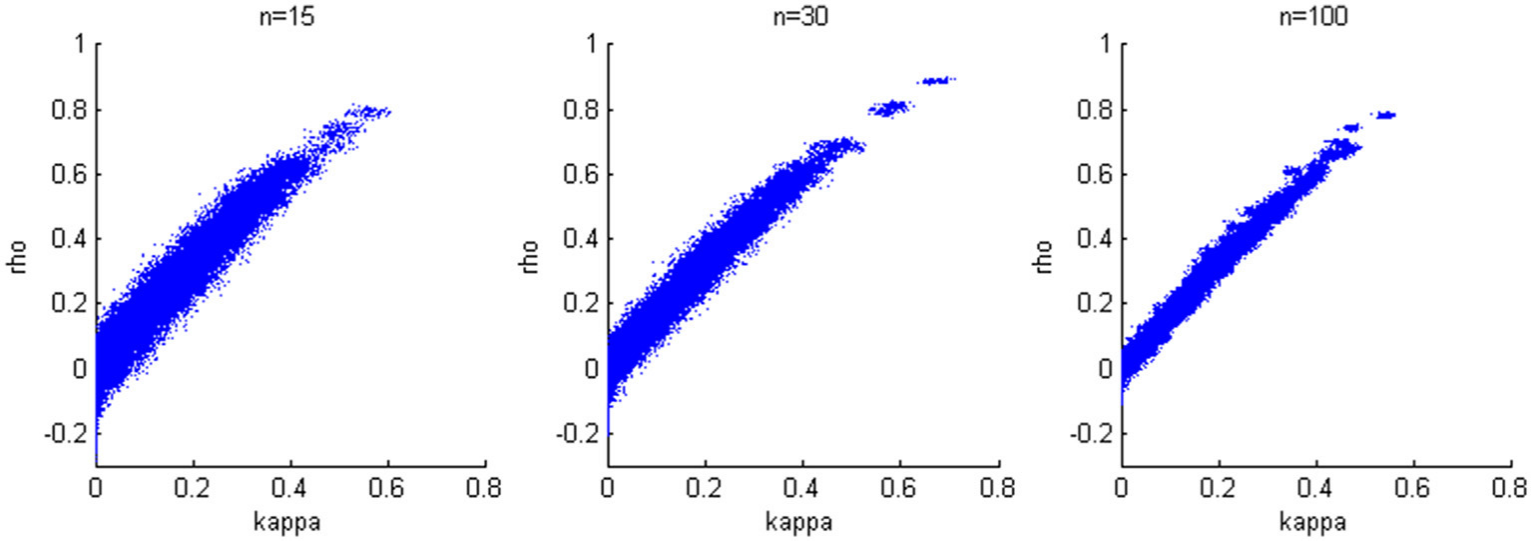
**Relationship between κ and ρ.** A positive linear relationship is detected for three cases with different sample sizes.

We generate results for all combinations of hyperparameters specified in the first simulation study and again consider sample sizes of 15, 30, and 100. The accuracy of the estimation is not heavily influenced by the variance of the bivariate normal distribution; therefore, we present results when σ^2^_*a*_ = σ^2^_*b*_ = 0.2, which corresponds to estimates from our experimental data. We find a positive linear relationship between κ and ρ (Figure 4), while larger sample size yields smaller variability in the estimates.

**Figure 5:**
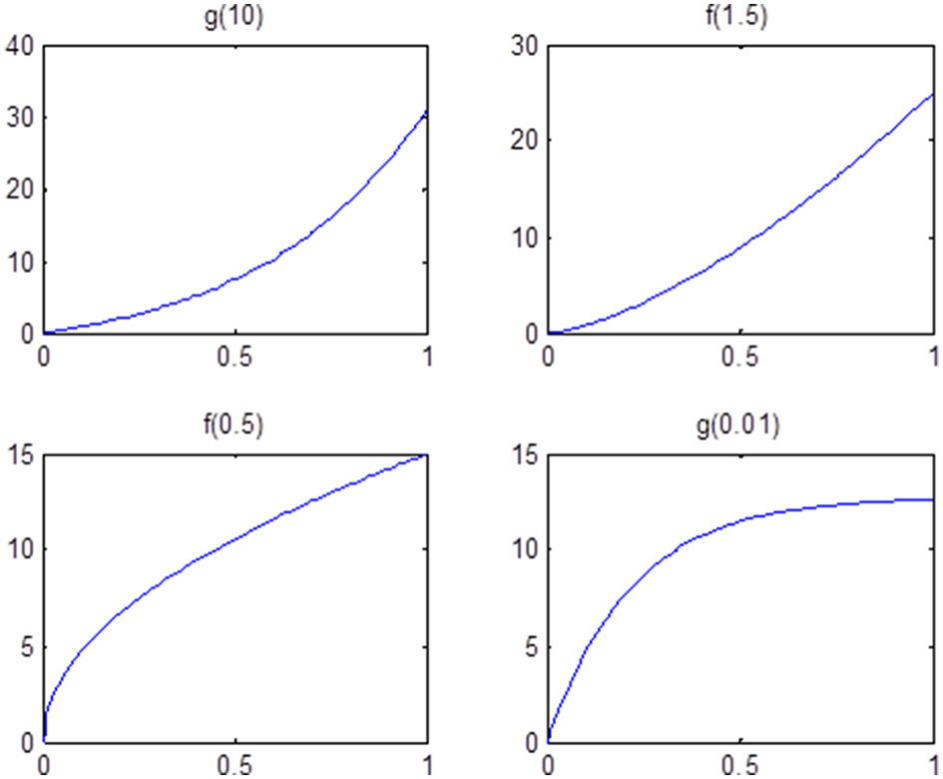
**Functions that are used in the sensitivity analysis of α(π).** All of them are increasing functions with respect to π and have the same area under curve.

Finally, we examine the impact of α(π) on the estimation of **θ**. As the data suggests, the functional coherence tends to increase as the SC increases. Therefore, we use four different increasing functions for the parameter α(π) as a parameter of the prior distribution of **θ**, based on power functions and exponential functions. Figure 5 shows the functions that we consider in the posterior simulations. As each function has the same integration over the interval [0, 1], the expected values of all the θ_1_'s simulated from each function are the same. We choose specific forms of functions *f* and *g* to satisfy the above criterion. We consider both convex functions, i.e., *g*(10) and *f*(1.5), and concave functions, i.e., *f*(0.5) and *g*(0.01). Table 3 summarizes the biases of **θ** estimated using different functions, where the true values are generated from the two most extreme cases *g*(10) and *g*(0.01). Here, the bias is calculated from the sum of the biases in all θ_*i*_'s. We also vary the probability of SC, π, from weak to strong in the simulation study. The results indicate that the biases of **θ** across all tested functions are comparable. Thus, we conclude that our method is not very sensitive to the choice of α(π), among those considered.

**Table 3:** **The bias of **θ** from estimations of different functions of α(π)**.

	**Generate Data from *g*(10)**					**Generate Data from *g*(0.01)**			
**π**	***g*(10)**	***f*(1.5)**	***f*(0.5)**	***g*(0.01)**	**π**	***g*(10)**	***f*(1.5)**	***f*(0.5)**	***g*(0.01)**
0.2045	0.0024	0.0026	0.0047	0.0051	0.0325	0.0018	0.0017	0.0023	0.0021
0.4626	0.0034	0.0035	0.004	0.0042	0.164	0.0035	0.0034	0.0026	0.0027
0.6894	0.0041	0.0038	0.0043	0.0043	0.4724	0.0038	0.0037	0.0035	0.0034
0.8987	0.0066	0.0073	0.0086	0.009	0.7031	0.0059	0.0058	0.0061	0.0059

*Note that not much difference is detected across different functions with the same structural connectivity. The bias is calculated from the sum of the bias in all θ_i_'s. Here*, *f*(*a*) = 10 ×(*a* + 1) × π^*a*^, $g(a)=10/\left(\frac{a-1}{\ln(a)}-1\right)\times a^{\pi }-10/\left(\frac{a-1}{\ln(a)}-1\right)$

## 5. Discussion

We build a unified Bayesian framework that provides a novel approach to combine functional and structural brain imaging for an integrated assessment of FC to evaluate the whole-brain networks. Joint analysis of both imaging modalities is an important tool to gain a better understanding of sensory and cognitive functions in the human brain as well as pathophysiology associated with psychiatric or neurologic disorders. Different from other methods that examine both FC and SC in a descriptive way, our method incorporates SC information into the model and allows for making statistical inferences, which acts as a basis of building a functional network. Our Bayesian model utilizes SC information to estimate the functional coherence between pairs of regions, yet our model does not allow the structural information to unduly drive the functional results. Our method is a purely data-driven, hypothesis-unconstrained approach, which can efficiently search across all pairs of defined brain regions of interest. We develop two measures, κ and τ, to capture the functional coherence and degree of ascendancy, between the brain regions. κ is based on the probability of joint activation and deactivation, leading to an undirected network; while τ assesses the ascendancy between functionally connected regions, enabling us to construct a directed brain network. By incorporating both FC and SC, we more accurately identify the hubs and reduce the noise in the brain network.

Our method permits analyses examining all 20910 possible brain region pairs, between 205 subregions, to construct FC networks. We conduct a whole-brain analysis rather than requiring a pre-defined network or regions of interest. Our method is based on subregions centered on the most active voxel, yielding neural activity profiles that are representative of the brain activity within the small spherical subregions. Unlike other methods that average data across an entire region, we generate neural activity summaries for each subregion to allow heterogeneous patterns across the whole region. The mean of the time course puts equal weight on each voxel, which may not be able to explain the variation across the subregion. Instead, we use the first singular vector from SVD to represent the subregion, which is a weighted combination of neural activity profiles, and provides additional flexibility to summarize the information. We may allow multiple subregions from each AAL region, which provides more complete coverage in our whole-brain analyses.

We dichotomize the time series data to define functional joint activations, from which we evaluate the functional connectivity using our proposed κ metric that differs from the traditional correlation approach. There is no scale for the fMRI signal in the human brain that lends itself to natural interpretations of the level of neural activity. We define high and low neural activity from the fMRI signal based on a selected threshold. A possible extension to our current method is to use finer categories such as ordinal or even continuous to define joint functional activations.

In the context of dichotomizing the data, our modeling framework depends on *c* to declare elevated and inactive states of neural activity. Drastic changes to *c* will have a direct impact on the data that are input to our model, e.g., by setting *c* sufficiently high (or low), all the regions will become inactive (or active). We choose an arbitrary value of *c* reflecting the threshold for a standard score of fMRI BOLD activity. A reasonable default threshold is *c* = 0 to designate increased or decreased activity relative to the mean level. We use a slightly elevated value of *c* = 0.01 when determining the indicator of elevated brain activity for regional fMRI profiles from our data, which results in about 45% of the time points being regarded as active states. We conduct sensitivity analysis of the choice of *c*, and find that when *c* fluctuates within a small range, i.e., for *c* up to 0.1, the major findings in our data application do not change.

We propose a functional coherence measurement that builds on Cohen's κ-statistic, which evaluates the levels of agreement adjusted for chance. Here, the chance agreement is defined as (θ_1_ + θ_2_)(θ_1_ + θ_3_) + (θ_3_ + θ_4_)(θ_2_ + θ_4_). In addition to Cohen's κ, we may consider other agreement measures, e.g., Scott's π-statistic (Scott, 1955), in which the chance agreement is obtained by [((θ_1_ + θ_2_) + (θ_1_ + θ_3_))/2]^2^ + [((θ_3_ + θ_4_) + (θ_2_ + θ_4_))/2]^2^; Fleiss' κ-statistic (Fleiss, 1971), which is a generalization of Scott's π; and other alternative chance-corrected statistics (Gwet, 2002). The major difference among these statistics is the way they calculate the chance agreement. Some researchers (Gwet, 2002) argue that the conditions that Cohen's κ requires, e.g., the chance-agreement probability is less than 0.5, are not always met in practice. In our case, however, the sum of the marginal probabilities *P*(*A*_*a*_ = 1) + *P*(*A*_*b*_ = 1) is close to 1, which ensures that the chance-agreement probability does not exceed 0.5. In addition, other statisticians (Strijbos et al., 2006) believe that when fewer categories are included, Cohen's κ is a more conservative measurement of agreement. Therefore, we use this more strict measurement in our case.

The study of functional networks in the human brain is important to understand basic cognition, mental and neurological disorders, and response to treatments for these disorders. Moreover, the structural circuitry underlying functional connections may offer additional insights. We develop a Bayesian model that combines both functional and structural information to help characterize FC networks. Leveraging SC to quantify FC, our model yields more accurate and more informative results than considering solely functional data.

### Conflict of interest statement

The authors declare that the research was conducted in the absence of any commercial or financial relationships that could be construed as a potential conflict of interest.
