# Supplementary material for: A multimodal approach for determining brain networks by jointly modeling functional and structural connectivity
Source: Front Comput Neurosci. 2015 Feb 20;9:22. doi: 10.3389/fncom.2015.00022 (PMC4335182; doi:10.3389/fncom.2015.00022)
Supplement: Supplementary file 1 [file DataSheet1.PDF]

Appendix

Appendix 1: Brain parcellation

We consider a brain parcellation that is a more granular version of the AAL regional template; we utilize the existing boundaries but provide a further subdivision within each region of interest. A hierarchical clustering algorithm is applied to each region, where the spatial contiguity of the resulting clusters is enforced using the distance matrix. Connectivity information, averaged across the sample, is utilized to subtly promote functional and structural homogeneity in the gray and white matter, respectively, of each resulting cluster. Finally, we incorporate tissue-type information (gray/white matter) in the distance matrix to encourage balance in the tissue composition of clusters. Based on average linkage, the number of clusters is chosen manually to yield a resulting parcellation consisting of approximately 200 regions, using the Hierarchical Clustering tools in MATLAB and Statistics Toolbox Release 2012b (The MathWorks, Inc., Natick, Massachusetts, United States). Independently for each AAL region, let d_{ij} represent the Manhattan distance, which is the sum of the absolute

differences of MNI coordinates, for voxels i and j, and define the distance matrix as:

$$D_{ij} = \begin{cases} d_{ij} + (1 - \mathrm{FC}_{ij}) & \text{if } i \in \mathcal{G}, j \in \mathcal{G} \\ d_{ij} + (1 - \mathrm{SC}_{ij}) & \text{if } i \in \mathcal{W}, j \in \mathcal{W} \\ d_{ij} & \text{otherwise,} \end{cases} \tag{13}$$

where FC_{ij} represents the average of functional connectivity for voxel pairs in gray matter $\mathcal{G}$ and SC_{ij} represents the average of structural connectivity for voxel pairs in white matter $\mathcal{W}$. Functional connectivity is quantified by estimating the Pearson correlation coefficient between the fMRI-derived BOLD signals of gray matter voxel pairs for each subject, and averaging across subjects. Structural connectivity is quantified by calculating the correlation between "connectivity profiles" and subsequently averaging across subjects. The "connectivity profile" for a white matter voxel is obtained by using a set of predefined target regions scattered throughout the brain and applying probabilistic tractography tools in FSL (Behrens, 2003) to each subject's DTI data, yielding values that reflect the strength of structural connectivity between the voxel and each of the target regions. Thus, SC_{ij} is expected to reflect the average consistency in structural connections between white matter voxel pairs. For tissue-heterogenous voxel pairs, there is no obvious way to quantify connectivity; by using an unpenalized distance of d_{ij} for these mismatched voxel pairs, the resulting clusters tend to span both gray and white matter.

Appendix 2: Definition of κ and τ in Patel et al. (2006a)

The functional connectivity κ is defined as:

$$\kappa = \frac{\theta_1 - E}{D(\max(\theta_1) - E) + (1 - D)(E - \min(\theta_1))}, \tag{14}$$

where $E = (\theta_1+\theta_2)(\theta_1+\theta_3)$, $\max(\theta_1) = \min(\theta_1+\theta_2, \theta_1+\theta_3)$, $\min(\theta_1) = \max(0, 2\theta_1+\theta_2+\theta_3-1)$, and

$$D = \begin{cases} \frac{\theta_1 - E}{2(\max(\theta_1)-E)} + 0.5, \text{ if } \theta_1 \geq E \\ 0.5 - \frac{\theta_1 - E}{2(E-\min(\theta_1))}, \text{ otherwise .} \end{cases} \tag{15}$$

The functional ascendancy τ takes the form:

$$\tau = \begin{cases} 1 - \frac{\theta_1+\theta_3}{\theta_1+\theta_2}, \text{ if } \theta_2 \geq \theta_3 \\ \frac{\theta_1+\theta_2}{\theta_1+\theta_3} - 1, \text{ otherwise.} \end{cases} \tag{16}$$

As noted in the manuscript, our definitions of κ and τ extend those presented in Patel et al. (2006a) by capturing coherence. This is reflected by incorporating θ_4 in the numerator of the definition of κ.

Appendix 3: A list of regions included in the directed network

Right anterior cingulate gyrus (AC_R)	Left angular gyrus (Ang_L)	Right angular gyrus (Ang_R)
Left Calcarine gyrus (Cal_L)	Right Calcarine gyrus (Cal_R)	Left caudate gyrus (Cau_L)
Right caudate gyrus (Cau_R)	Left fusiform gyrus (Fus_L)	Left Heschl gyrus (Hes_L)
Left Hippocampus (Hipp_L)	Left Insular gyrus (Ins_L)	Right Insular gyrus (Ins_R)
Left inferior occipital gyrus (IO_L)	Left inferior temporal gyrus (IT_L)	Left lingual gyrus (Ling_L)
Right lingual gyrus (Ling_R)	Right middle cingulate gyrus (MC_R)	Left middle frontal gyrus (MF_L)
Right middle frontal gyrus (MF_R)	Left middle occipital gyrus (MO_L)	Left medial orbital frontal gyrus (MOF_L)
Right medial orbital frontal gyrus (MOF_R)	Left middle temporal gyrus (MT_L)	Right middle temporal gyrus (MT_R)
Left olfactory gyrus (OLF_L)	Left pallidum (Pal_L)	Right Parahippocampal gyrus (ParaHipp_R)
Left Precentral gyrus (PC_L)	Right Precentral gyrus (PC_R)	Left paracentral lobule (PL_L)
Right paracentral lobule (PL_R)	Right middle temporal pole (PMT_R)	Left postcentral gyrus (PoC_L)
Right postcentral gyrus (PoC_R)	Left superior temporal pole (PST_L)	Right superior temporal pole (PST_R)
Left putamen gyrus (Put_L)	Left gyrus Rectus (Rec_L)	Left Rolandic operculum (RO_L)
Right Rolandic operculum (RO_R)	Left superior frontal gyrus (SF_L)	Right superior frontal gyrus (SF_R)
Left superior medial front gyrus (SMF_L)	Right superior medial front gyrus (SMF_R)	Left superior occipital gyrus (SO_L)
Left superior tempeoral gyrus (ST_L)	Right superior tempeoral gyrus (ST_R)	Right Thalamus (Thal_R)

Appendix 4: Component figures for each driven hub shown in Figure 3

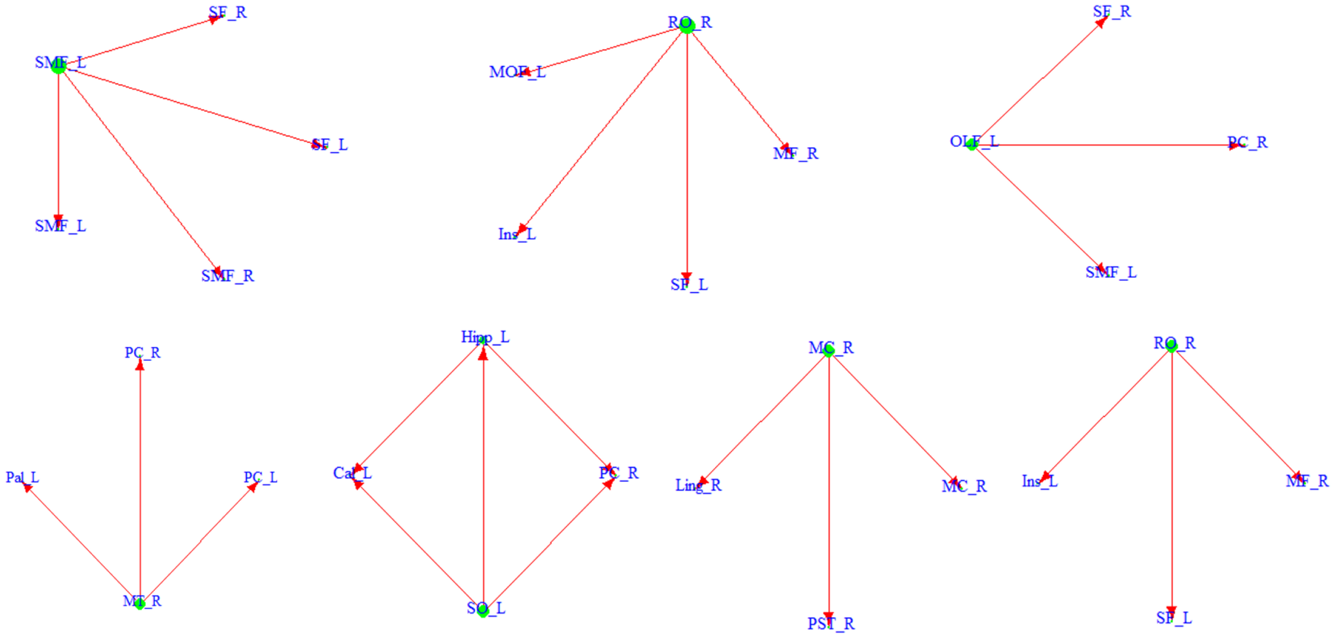

Appendix 5: Simulation results

Table 4: Comparison of mean of bias between two Bayesian methods. The table reveals the improvements of FC with SC from FC only in terms of the standard deviation of bias.

			FC with SC ($\times 10^{-3}$)						*FC only* ($\times 10^{-3}$)					
α_0	β_0	$E(\pi)$	θ_1	θ_2	θ_3	θ_4	κ	τ	θ_1	θ_2	θ_3	θ_4	κ	τ
N=15														
1	100	0.01	5.457	6.792	6.827	6.954	8.146	36.287	5.536	6.890	6.952	7.011	170.042	65.855
2	18	0.1	5.783	6.945	6.757	6.852	10.037	34.913	5.854	6.976	6.869	6.936	166.338	70.194
2	5	0.3	6.576	6.733	6.537	6.431	13.389	33.654	6.610	6.824	6.633	6.502	122.500	70.714
2	2	0.5	7.072	6.271	6.575	6.482	14.457	33.382	7.131	6.385	6.704	6.594	78.412	75.823
5	2	0.7	7.339	6.267	6.200	6.253	15.060	32.658	7.407	6.354	6.268	6.393	44.905	78.162
18	2	0.9	7.649	5.681	5.792	5.631	15.049	32.852	7.762	5.807	5.886	5.728	51.656	90.954
N=30														
1	100	0.01	3.817	4.930	4.889	4.906	6.049	25.263	3.857	4.950	4.911	4.944	191.969	64.374
2	18	0.1	4.022	4.817	4.806	4.961	7.070	24.206	4.066	4.851	4.827	4.994	160.240	62.968
2	5	0.3	4.581	4.730	4.767	4.772	9.269	24.365	4.600	4.757	4.806	4.783	131.978	69.942
2	2	0.5	4.806	4.602	4.620	4.636	10.160	24.665	4.858	4.655	4.647	4.669	113.153	76.524
5	2	0.7	5.277	4.381	4.375	4.411	10.745	23.596	5.340	4.417	4.412	4.438	45.638	80.886
18	2	0.9	5.411	4.133	4.060	4.183	10.798	22.775	5.481	4.171	4.105	4.213	48.692	88.015
N=100														
1	100	0.01	2.148	2.726	2.753	2.680	3.299	14.407	2.156	2.734	2.758	2.688	183.179	64.847
2	18	0.1	2.315	2.683	2.637	2.691	4.586	13.796	2.318	2.687	2.640	2.694	157.503	67.204
2	5	0.3	2.428	2.628	2.631	2.589	4.949	13.279	2.436	2.632	2.636	2.596	129.687	69.334
2	2	0.5	2.729	2.560	2.500	2.570	5.964	13.173	2.738	2.564	2.502	2.583	109.046	76.622
5	2	0.7	2.817	2.440	2.430	2.354	5.991	12.225	2.821	2.442	2.433	2.359	64.481	79.986
18	2	0.9	2.992	2.274	2.337	2.248	6.085	13.011	3.001	2.282	2.347	2.257	48.929	87.404
